# Responses to Social Vocalizations in the Dorsal Cochlear Nucleus of Mice

**DOI:** 10.3389/fnsys.2015.00172

**Published:** 2015-12-16

**Authors:** Patrick D. Roberts, Christine V. Portfors

**Affiliations:** School of Biological Sciences and Integrative Physiology and Neuroscience, Washington State UniversityVancouver, WA, USA

**Keywords:** dorsal cochlear nucleus, mouse, frequency tuning, vocalizations, distortion products

## Abstract

Identifying sounds is critical for an animal to make appropriate behavioral responses to environmental stimuli, including vocalizations from conspecifics. Identification of vocalizations may be supported by neuronal selectivity in the auditory pathway. The first place in the ascending auditory pathway where neuronal selectivity to vocalizations has been found is in the inferior colliculus (IC), but very few brainstem nuclei have been evaluated. Here, we tested whether selectivity to vocalizations is present in the dorsal cochlear nucleus (DCN). We recorded extracellular neural responses in the DCN of mice and found that fusiform cells responded in a heterogeneous and selective manner to mouse ultrasonic vocalizations. Most fusiform cells responded to vocalizations that contained spectral energy at much higher frequencies than the characteristic frequencies of the cells. To understand this mismatch of stimulus properties and frequency tuning of the cells, we developed a dynamic, nonlinear model of the cochlea that simulates cochlear distortion products on the basilar membrane. We preprocessed the vocalization stimuli through this model and compared responses to these distorted vocalizations with responses to the original vocalizations. We found that fusiform cells in the DCN respond in a heterogeneous manner to vocalizations, and that these neurons can use distortion products as a mechanism for encoding ultrasonic vocalizations. In addition, the selective neuronal responses were dependent on the presence of inhibitory sidebands that modulated the response depending on the temporal structure of the distortion product. These findings suggest that important processing of complex sounds occurs at a very early stage of central auditory processing and is not strictly a function of the cortex.

## 1. Introduction

An important task of neuroscience is to understand the multiple processing stages of behaviorally important sensory stimuli. The initial processing of sensory stimuli begins at the transduction to neural signals at the periphery, and then behaviorally relevant information is filtered at each subsequent nucleus within the sensory pathway. Presently, our understanding of the nervous system has confirmed that no nucleus is a simple relay (Coombs et al., 1998; Casseday et al., 2002; Sherman, 2007), but rather each nucleus filters and transforms information in ways that depend on its intrinsic properties, morphology, and circuitry. Moreover, features that were previously thought to be extracted at high-level processing centers in cortical regions are now known to be extracted subcortically. Thus, it is important to understand how behaviorally relevant stimuli are encoded at multiple stages along the central sensory pathways.

The encoding of complex sounds such as vocalizations has historically been considered a function of the auditory cortex as cortical neurons are selective for both spectral and temporal features of species-specific vocalizations (Wollberg and Newman, 1972; Glass and Wollberg, 1983; Wang et al., 1995; Wang and Kadia, 2001). These neurons often respond better to vocalizations than pure tones and/or their responses to vocalizations cannot be predicted by their responses to pure tones. In addition, neurons with similar excitatory receptive fields respond differently to a number of different vocalizations. However, when responses to vocalizations have been examined in subcortical structures, in many cases, similar levels of selectivity have been found (Portfors and Wenstrup, 1999; Klug et al., 2002; Portfors et al., 2009; Holmstrom et al., 2010; Portfors and Roberts, 2014). In particular, responses in the main auditory midbrain nucleus, the inferior colliculus (IC), have been found to be selective to spectral and temporal features of social vocalizations of bats (Klug et al., 2002; Portfors, 2004) and mice (Portfors et al., 2009; Holmstrom et al., 2010). Similar to responses in the cortex, the responses to vocalizations in the IC are not well predicted by excitatory receptive fields and there is heterogeneity in the way neurons with similar frequency tuning respond to the same suite of vocalizations (Klug et al., 2002; Holmstrom et al., 2010).

In contrast, neurons in the nuclei of the lateral lemniscus (NLL), a brainstem region that projects to the IC, are not selective to vocalizations (Bauer et al., 2002; Xie et al., 2005). These neurons respond in a homogeneous manner to a suite of vocalizations and their responses are well predicted by their excitatory frequency tuning curves. The explanation for the differences in responses in the IC and NLL is that neurons in the IC are strongly innervated by inhibitory inputs that play a role in shaping receptive fields and creating selective responses to vocalizations (Xie et al., 2005; Mayko et al., 2012). Based on the homogeneous responses and lack of selectivity to vocalizations in the NLL, it has been suggested that selectivity to vocalizations emerges in the IC due to the complex interplay between excitation and inhibition in this structure (Bauer et al., 2002; Xie et al., 2005; Pollak, 2013). However, responses to vocalizations and levels of selectivity have not been extensively tested in other brainstem nuclei and in particular, they have not been tested in structures that also have strong inhibition that could shape the way neurons respond to complex sounds. In this study, we examined whether neurons in the dorsal cochlear nucleus (DCN), one of the first synapses in the central auditory pathway, respond in a heterogeneous and selective manner to vocalizations.

The DCN is an auditory brainstem nucleus where significant transformations of sensory information take place (Yu and Young, 2000). It is a cerebellum-like structure that integrates direct ascending information from the auditory nerve with descending multimodal inputs (Oertel and Young, 2004). Fusiform cells, the neurons that project to the IC, receive multiple excitatory and inhibitory inputs that could shape receptive fields to create heterogeneous and selective responses to vocalizations. Fusiform cells receive excitatory input from auditory nerve afferents and from parallel fibers that convey information from a wide range of auditory and non-auditory sources (Brown et al., 1988; Golding et al., 1995; Weedman and Ryugo, 1996; Shore et al., 2000). They also receive inputs from inhibitory interneurons; vertical cells, cartwheel cells, and stellate cells. The vertical cells receive direct auditory nerve input and provide lateral inhibition to fusiform cells. Cartwheel cells and stellate cells receive inputs from parallel fibers and provide inhibitory input that may be complex in its frequency tuning (Portfors and Roberts, 2007; Roberts and Portfors, 2008). Thus, the variety of excitatory and inhibitory inputs lead to the prediction that fusiform cells will respond to vocalizations in a heterogeneous manner. We tested this prediction in the DCN of awake mice.

This prediction however is somewhat complicated by the fact that the social vocalizations often emitted by mice contain energy at frequencies that are much higher than those most represented in the mouse auditory system (Portfors et al., 2009; Woolley and Portfors, 2013; Portfors and Roberts, 2014). Previous studies in the IC of mice have suggested that neurons with low frequency tuning curves respond to these ultrasonic vocalizations because of distortion products (Portfors et al., 2009; Portfors and Roberts, 2014) and this hypothesis has been further supported by fMRI studies in the rat IC (Gao et al., 2015). Because distortion products are produced in the cochlea when multiple signals with different frequencies occur simultaneously, we expect that neurons in DCN should also respond to distortion products and be used as a mechanism for encoding ultrasonic vocalizations in the mouse.

By recording responses of fusiform cells to vocalizations in the DCN of mice, we demonstrate in this study that the output neurons of the DCN respond in a heterogeneous and selective manner to vocalizations. In addition, we found that fusiform cells can use distortion products as a mechanism for encoding ultrasonic vocalizations. These findings suggest that important processing of complex sounds occurs at a very early stage of central auditory processing and is not strictly a function of the cortex.

## 2. Materials and methods

We recorded single unit responses to simple stimuli and vocalizations in the DCN of awake mice.

### 2.1. Animals

Seventy-two CBA/CaJ mice (46 female, 26 male), 2–6 months, were used in this study. The animals were housed with same sex littermate pairs until the surgery for electrophysiological recordings. The mice were housed under a reversed 12 h light/dark cycle and electrophysiological recordings were performed during their awake period. Food and water were provided *ad libitum*. All animal care and experimental procedures were in accordance with the guidelines of the National Institutes of Health and approved by the Washington State University Institutional Animal Care and Use committee.

### 2.2. Surgical procedures

To immobilize the head for single unit recordings in the DCN, we attached a metal pin to the skull and bolted the pin to a custom-made stereotaxic apparatus (Muniak et al., 2012). During the surgery to attach the headpin, the animal was anesthetized with isoflurane inhalation and restrained in a stereotaxic frame with earbars to secure the head. Care was taken to avoid damage to the tympanic membrane. Briefly, a midline incision was made in the skin and it was removed on the left side to expose the region of the skull dorsal to the DCN. Ultraviolet-cured dental cement was used to cement the pin to the skull and a tungsten ground electrode was cemented into the right cerebral cortex. A craniotomy was made to expose the cerebellum and a portion of the IC using stereotaxic coordinates from the mouse brain atlas (Paxinos and Franklin, 2001): between 5.6 and 6.3 mm caudal to the bregma line and between 2.0 and 2.6 mm from the midline. After the surgery, a local anesthetic (lidocaine) and a topical antibiotic (Neosporin) were applied to the incision and the animal was placed in an isolated cage for 1–2 days before electrophysiological recordings.

### 2.3. Electrophysiological recording procedure

The awake animal was restrained in a molded piece of foam with the headpin secured in the stereotaxic apparatus. The animal was initially sedated with a light dose of acepromazine (5 mg/kg, i.p.) to ease the process of restraining the animal. The custom stereotaxic apparatus was on an air table that was located in a single-walled sound-attenuation chamber covered internally with acoustical foam. Unless the animal struggled excessively, the recording sessions lasted 4–5 h. Recordings were performed on the same animal on 1–3 separate days. Between recording sessions, the craniotomy was covered with bone wax and the mouse was housed individually.

Well-isolated single-unit responses were obtained with glass micropipettes filled with 1M NaCl (resistance was 15–30 MΩ). Recording electrodes were advanced by a hydraulic micropositioner (model 650D; David Kopf Instruments) driven from outside the sound attenuating chamber. Extracellular electrical activity was amplified (model 2400; Dagan), filtered (band-pass, 500–6000 Hz; model 3364; Krohn-Hite), and sent through a spike enhancer (FHC) before being digitized (10,000 samples/s; Microstar Laboratories), displayed and then stored using custom data acquisition software. Waveforms, raster plots, and histograms of spike responses were visualized on-line during recordings, and then stored for off-line analysis using custom routines written in Matlab (The MathWorks, Natick, MA) and Python (Python Software Foundation, Python.org).

The dorsal extent of the DCN was located approximately 2.7 mm below the surface of the cerebellum. We confirmed that our recording electrodes were consistently locating the DCN in several animals using iontophoretic deposits of dextran conjugated rhodamine (Portfors and Roberts, 2007). Once we isolated a single unit such that the extracellular action potentials clearly extended above the background activity with a signal-to-noise ratio of at least 2:1 but most frequently 4:1, we presented a series of acoustic stimuli to identify the cell-type and investigate responses to natural vocalizations.

### 2.4. Acoustic stimulation

Acoustic stimuli [pure tones, broad band noise, or mouse vocalizations synthesized from natural recordings using custom software (Holmstrom et al., 2009)], were presented using custom software. Stimuli were converted to an analog signal with a high-speed, 16-bit D/A converter (400,000 samples/s; Microstar Laboratories), filtered though a programmable attenuator (model PA5; Tucker Davis Technologies), sent to a power amplifier (model HCA-1000A; Parasound), and presented from a leaf tweeter speaker (Infinity) located 10 cm away from the mouse. The properties of the acoustic presentation system were regularly tested using a 1/4-in. calibrated microphone (model 4135, Brüel & Kjær) placed in the position normally occupied by the animal's ear. Both the sound pressure level (SPL) and the spectral characteristics were tested to characterize the effect of stimulus frequency on SPL and to identify (and eliminate) the presence of any possible spectral distortions. There was a monotonic decrease in sound pressure from 6 to 60 kHz of about 2.7 dB per 10 kHz. Distortion components were in the noise floor, <50 dB below the signal level, as measured by custom software using a fast Fourier analysis of the microphone signal.

We used both pure tones (8–50 kHz, 40–80 dB SPL) and broadband noise (BBN, 40–80 dB SPL) as our search stimuli. When a single unit was isolated, we audiovisually found characteristic frequency (CF) and threshold, and often found the BBN threshold to aid in identifying the cell type. We then captured the spike response to the CF (50 ms duration) with 20 repetitions at 10 dB above the CF threshold intensity. All acoustic stimuli were presented at a repetition rate of 3 Hz. We collected pure tone responses to obtain frequency tuning curves (8–100 kHz, 4 kHz steps, 1–3 SPLs including 10 dB above CF threshold). For many units, we also tested for pure tone responses presented at the level of the vocalizations (40–100 kHz, 2 kHz steps, 65 dB SPL). The responses to BBN were tested at a range of intensities (10–80 dB SPL) to determine the BBN threshold.

We then presented a suite of 35 mouse vocalizations at 65 dB SPL for 20 repetitions each. The vocalizations were chosen from our database of natural mouse vocalizations emitted by male mice during social interactions (Mahrt et al., 2013). These vocalizations were chosen because they represent the most commonly emitted syllable types of males during courtship behaviors and the spectral energy of all the stimuli was far higher than the CFs of the majority of cells in the DCN. The spectral content of all vocalizations used in this study are shown in the Results. The minimal low frequency noise was –75dB below the maximum stimulus intensity.

### 2.5. Identification of cell types in DCN based on electrophysiological responses

Extensive *in vitro* and *in vivo* experiments in DCN have established that particular cell types respond to sound in specific manners (see Young and Davis, 2002). These physiological response types are distinguishable in extracellular neural recordings and provide a basis for identifying cell types *in vivo*. We applied the same criteria as in our previous studies (Portfors and Roberts, 2007; Roberts and Portfors, 2008) to associate the morphological cell type with electrophysiological characteristics. This study focuses entirely on fusiform cells. Fusiform cells are associated with both type III and type IV responses (Young and Davis, 2002) although Type IV responses have not been observed in mouse (Portfors and Roberts, 2007; Roberts and Portfors, 2008) or gerbil (Parsons et al., 2001). Type III responses have V-shaped tuning, sideband inhibition, high rates of spontaneous activity, and good responses to broadband noise.

We could differentiate fusiform cells from cartwheel cells because of the presence of complex spikes in cartwheel cells (Zhang and Oertel, 1993; Manis et al., 1994; Parham and Kim, 1995) and their long latency responses to auditory stimuli. We could differentiate fusiform cells from vertical cells because vertical cells have thresholds to broadband noise that are less than 1/3 of the threshold to pure tones and they have narrow tuning curves (Young and Brownell, 1976; Voigt and Young, 1980; Davis et al., 1996a; Davis and Young, 1997; Roberts and Portfors, 2008). We were able to localize our recordings to the DCN rather than the ventral cochlear nucleus (VCN) through a combination of known recording depths for DCN responses in mouse (Portfors and Roberts, 2007) and a reversal in tonotopy from DCN to VCN.

### 2.6. Data analysis

The raw electrophysiological recording signals were examined off-line to ensure only well-isolated single units were included in this study. Spike times were extracted and stored for construction of histograms of responses to stimuli and frequency tuning curves.

Because many neurons in the DCN of awake mice were spontaneously active, evoked responses to acoustic stimulation were not well characterized by spike count alone. This ambiguity was because many responses involved an increase in spike rate followed by a decrease such that the total spike rate during the recording interval was not significantly affected. Therefore, we investigated statistical tests that could determine whether a spike pattern significantly deviated from a spontaneous rate pattern during the recording window. Comparisons with Poisson distributed spike trains could statistically distinguish responses (Brown et al., 2002), but these methods are more appropriate for long spike trains and many DCN neurons fire spontaneously in non-Poisson patterns. As an alternative, a non-parameteric statistical test was attempted for the peristimulus time histogram using a Kolmogorov-Smirnov (KS) test to determine whether there was a significant change from a constant spike pattern. However, our preliminary results suggested that several visually obvious responses were not statistically significant by this method because multi-phasic responses would hide the magnitude of the deviation.

To overcome these obstacles, we developed a method based on Bayesian statistics that identifies a time interval when a deviation of the spike rate has occurred during the stimulus cycle, and then estimates the probability of that deviation given the available data. This method applies Bayesian techniques to construct a statistical model of the spike data and estimate the distribution of parameters for spike rate and response timing.

The purpose of this analysis was to determine the probability that a stimulus causes a deflection in the spike probability during an interval of the stimulation cycle, and to quantify the effect size of the response. A significant deflection implied that the recorded neuron responded to the stimuli within the recording cycle.

We constructed a simple response model with four parameters, one parameter for the response latency (τ_1_), one for the duration (τ_2_), and two parameters for the two rates of activity: outside the response interval (λ_1_) and inside the interval (λ_2_). The advantage of this four-parameter model is that it can estimate whether a deviation occurred in the average spike rate anytime during the stimulus cycle. If the response is multi-phasic, then only one of the phases will be captured by this model, but the additional phases will not detract from the overall probability of a response.

To estimate the probability of a response to a stimulus, we applied a Monte Carlo Markov chain technique that is popular in Bayesian estimation statistics (Smith and Roberts, 1993). By considering the model parameters as random variables, θ = {λ_1_, λ_2_, τ_1_, τ_2_}, we calculated the probability of observed data, *D*, with the probability function, *P*(*D*|θ). However, we were interested in determining how the observed data constrained the parameters, *P*(θ|*D*). This Bayesian inversion of the conditional probability determined whether λ_2_, the magnitude of the response, significantly differed from λ_1_, the spontaneous activity rate.

**Figure 1:**
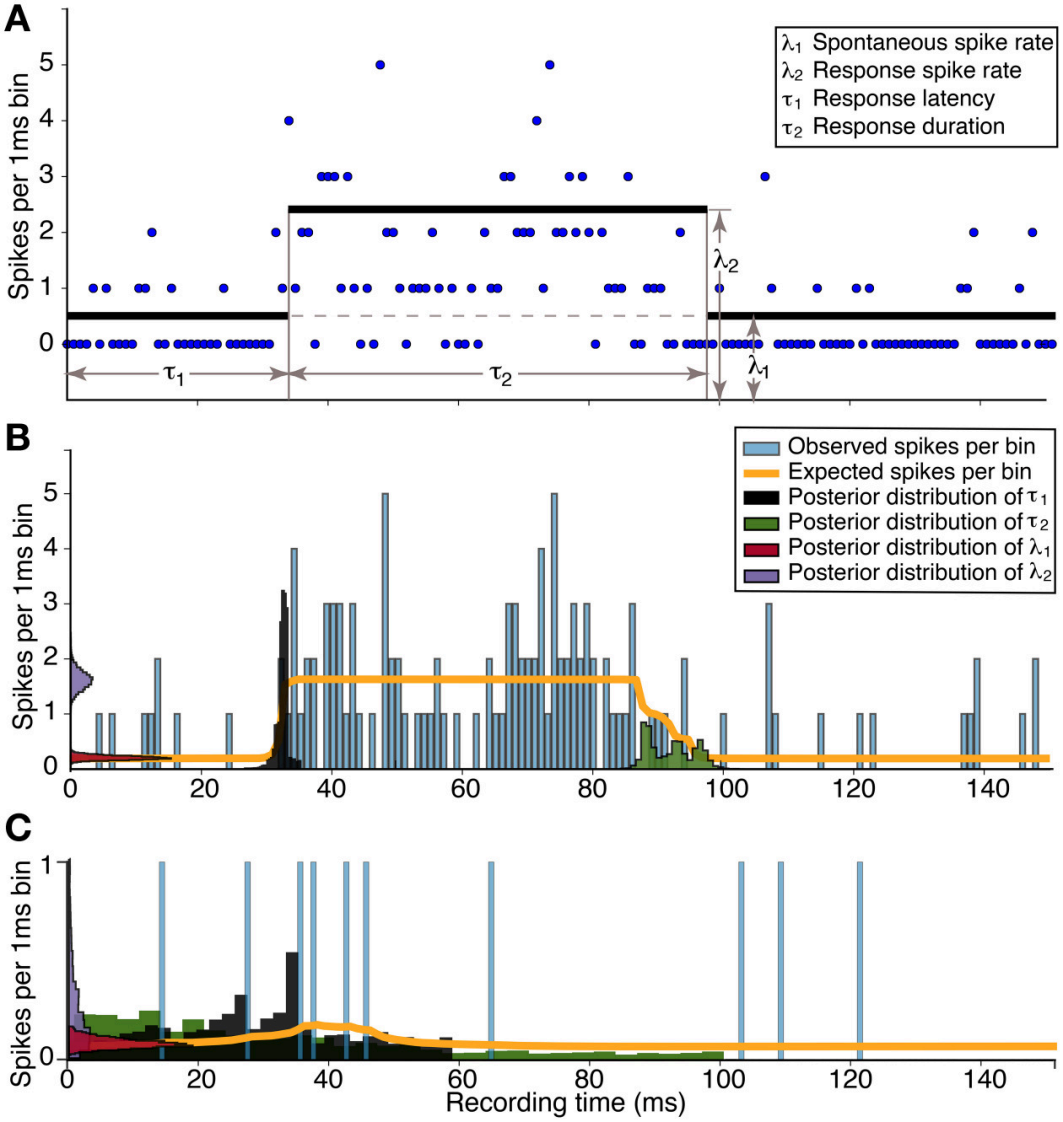
**Estimation of response parameters for a neuronal response to a tone. (A)** The data (blue dots) are spike times of 20 stimulus repetitions in 1 ms bins. Four parameters in the model represent the spontaneous spike rate (number of spikes per bin) of the neuron (λ_1_), the spike rate during the response (λ_2_), the onset latency of the response (τ_1_), and the duration of the response (τ_2_). **(B)** Histogram of the significant empirical response and the estimation; Response probability = 1.0, Magnitude = 1.44 ± 0.21, Latency = 32.29 ± 0.81 ms. **(C)** Histogram of the non-significant response and the estimation; Response probability = 0.65, Magnitude = 0.02 ± 0.29, Latency = 33.69 ± 14.60 ms. There is no overlap between the posterior distributions in **(B)** resulting in a response probability of 1.0. In contrast, the large overlap in **(C)**, in addition to the broad uncertainty of the latency, demonstrates the response probability below the significance level of 0.95.

To calculate the probability distributions of the model parameters constrained by the data, we applied the Metropolis-Hastings algorithm (Metropolis et al., 1953; Hastings, 1970). Two example response estimations are shown in Figure 1 for data collected from two single neurons in response to 20 presentations of a CF tone at 10 dB above threshold. The Metropolis-Hastings algorithm performs a random walk in parameter space that is constrained by the histogram data to find the probability distribution of parameter values. In the example in Figure 1B, there is no overlap between the distributions for λ_1_ and λ_2_. Therefore, there is a 100% probability of a response. Figure 1C illustrates a non-response because there was significant overlap.

When the model parameters were not well-constrained by the data, such as when there was a weak response to a stimulus, there may be considerable overlap between the probability distributions. We calculated the Hellinger distance (Costa et al., 2013),

(1) $$HD(P(\lambda_{1}),P(\lambda_{2}))=\sqrt{\frac{1}{2}\sum_{i\;=\;1}^{N}{\left(\sqrt{P_{i}(\lambda_{1})}-\sqrt{P_{i}(\lambda_{2})}\right)}^{2}},$$

where *N* is the number of bins, and *P*_*i*_(λ_*a*_) is the *i*-th bin of the estimated probability densitity of λ_*a*_ from the Metropolis-Hastings algorithm. The Hellinger distance becomes smaller as the overlap of the two probability distributions increases. To test whether there was a response to the stimulus (Basu et al., 2010), we set a significance level of a response that required *HD*(*P*(λ_1_), *P*(λ_2_)) ≥ 0.95.

### 2.7. Selectivity index

To quantify the amount of response selectivity for neurons that responded to at least one vocalization, we calculated the selectivity index (SI). The SI was calculated as SI = (*C*_*t*_ − *C*_*e*_)∕*C*_*t*_ where *C*_*t*_ was the number of vocalizations presented and *C*_*e*_ was the number of vocalizations that evoked a response, such that high index values indicated high selectivity. The SI was calculated at the same intensity for all neurons and all vocalizations.

### 2.8. Cochlea response model

Previous studies have suggested that auditory responses to mouse vocalizations in the IC are driven by distortion products generated by the cochlea (Portfors et al., 2009; Portfors and Roberts, 2014). To estimate the effects of cochlear distortions on responses to vocalizations in the DCN, we developed a phenomenological model of cochlear transduction consisting of two stages: a reverberation stage (Henson et al., 1995; Xie and Henson, 1998) and a distortion stage (Frank and Kössl, 1996). The reverberation stage is applied to the waveform of the vocalization and repeatedly adds the waveform to itself with a 1 ms delay, and each reverberation is reduced by an exponentially decreasing scaling factor.

(2) $$s^{\prime }(t)=\sum_{n\;=\;0}^{N}s(t-n\Delta t)e^{-n\Delta t/a}$$

where Δ*t* = 1 ms, *a* = 2 ms (Henson et al., 1995; Xie and Henson, 1998), *N* = 5. The final waveform was normalized to the original maximum amplitude.

To generate distortion products, we applied a Boltzman model (Frank and Kössl, 1996) to the signal following reverberation,

(3) $$D(x)=\frac{1}{1+\exp(a_{2}(x_{2}-x))(1+\exp(a_{1}(x_{1}-x)))}$$

where *x*_1_ = *x*_2_ = −0.2, *a*_1_ = 12.8, and *a*_2_ = *a*_1_∕3.

## 3. Results

We recorded auditory response properties of 160 single units in the DCN of awake mice to pure tones, broadband noise (BBN), and synthesized versions of mouse ultrasonic vocalizations. Eighty four of these recordings were identified as fusiform cells by their response properties to pure tones and broadband noise. Of these 84 fusiform cells, 48 responded to at least one of the vocalization stimuli. We focused our analysis on these 48 fusiform cells.

### 3.1. Fusiform cells have heterogeneous responses to vocalizations

**Figure 2:**
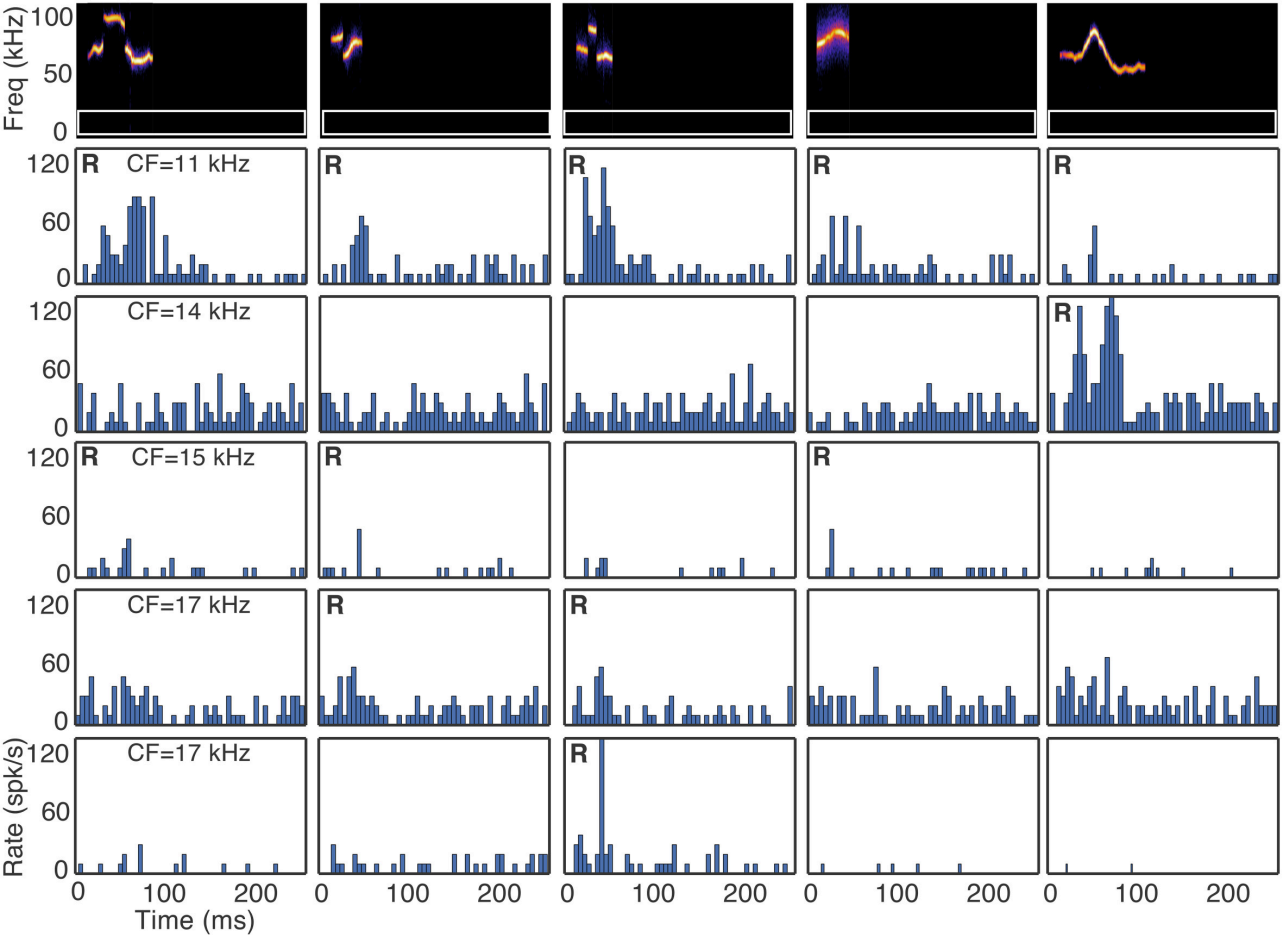
**Selectivity to vocalizations occurs in the DCN**. Responses of five fusiform cells (rows) to five vocalizations (columns) are shown. **R** indicates a statistically significant response to the vocalization. CF indicates the characteristic frequency of the cell. The white rectangular outlines in the spectrograms represent the excitatory frequency tuning of the neurons.

Fusiform cells responded in a heterogeneous manner to the suite of vocalizations. Figure 2 shows responses of five fusiform cells to five vocalization stimuli. A response to each vocalization, as determined by our Bayesian statistical criteria (Hellinger distance ≥ 0.95 for response magnitude relative to background spiking), is marked with an **R** in the upper left corner of the histogram panels. There are some histograms with visually apparent, weak responses, but the number of spikes could not be differentiated from noise with our sample size (20 stimulus repetitions) and were deemed non-significant by our statistical criteria.

**Figure 3:**
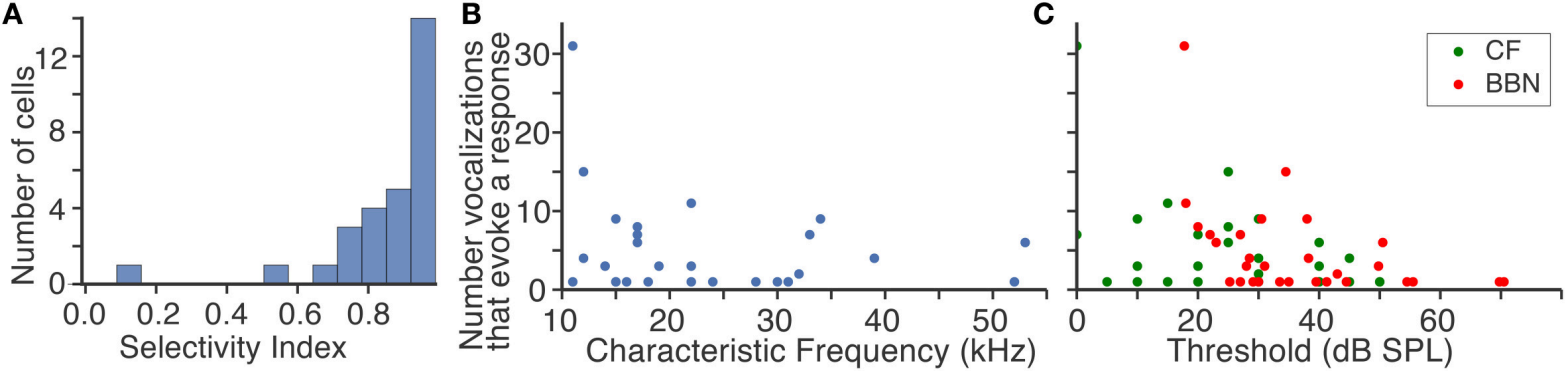
**Response characteristics of fusiform cells to vocalizations. (A)** Fusiform cells were highly selective to vocalizations. The median selectivity index value was 0.91. **(B)** There was no relation between the characteristic frequency of each neuron and how many vocalizations evoked responses. Each point is a fusiform cell. **(C)** Fusiform cells with high thresholds to a characteristic frequency tone and broad band noise were less likely to respond to vocalizations. Few cells with thresholds above 55 dB responded to vocalizations.

Two results are apparent from the example responses shown in Figure 2. First, fusiform cells responded differently to the suite of vocalizations even when the CFs of the neurons were similar. The selectivity index values (SI) indicate that fusiform cells were typically highly selective (Figure 3A). The median SI was 0.91, indicating that half of the cells responded to fewer than 10% of the vocalizations we presented. Although the fusiform cells were highly selective, the responses were distributed among the vocalization stimuli. At least one cell responded to each vocalization presented, and the maximum number of cells that responded to a single vocalization was 11. The mean number of cells that responded to each vocalization was 5.35±2.36. If the responses to vocalizations could be explained by a simple threshold effect, then we would expect that responses of cells with similar CFs would not be as widely distributed among the vocalizations as we have observed. The heterogeneous responses of fusiform cells to different vocalizations suggest that the population of fusiform cells projecting from the DCN can uniquely identify specific sounds.

The second result is that the CFs of the fusiform cells were strikingly different from the spectral energy of the vocalizations that evoked responses. The majority of CFs were below 20 kHz yet the spectral energy of the vocalizations that evoked responses was in the range of 50–100 kHz. This mismatch between neuronal frequency tuning and spectral energy of vocalizations has been examined in the IC (Portfors et al., 2009; Portfors and Roberts, 2014), and these neural responses were similar to those recorded here in the DCN. The CF of each fusiform cell was not correlated (*r*^2^ = 0.05) with the number of vocalizations with power in the 50–100 kHz range that evoked a response (Figure 3B). The sensitivity of the fusiform cells to stimuli, as measured by their minimal response thresholds to pure tones at their CF and broadband noise (Figure 3C), was only slightly correlated with the number of vocalizations that evoked a response (CF threshold, *r*^2^ = 0.17, BBN threshold, *r*^2^ = 0.19; Figure 3C). Therefore, the responses were independent of each cell's sensitivity to tones or broadband noise.

However, we did find a significant difference in sensitivity between the cells that responded to vocalizations and those that did not. The mean CF response threshold for the responders was 25.69±14.38 dB SPL compared to 41.19±16.98 dB SPL for the non-responders, (*p* < 10^−4^). There was also a significant difference between the mean BBN response thresholds for the responders (36.38±13.94) compared to the non-responders (60.97±14.98; *p* < 10^−9^). Therefore, some of the fusiform cells that did not respond to any vocalizations in our stimulus set might have responded if the vocalizations were presented at a higher intensity.

### 3.2. Cochlear distortion products can contribute to DCN responses to ultrasonic vocalizations

**Figure 4:**
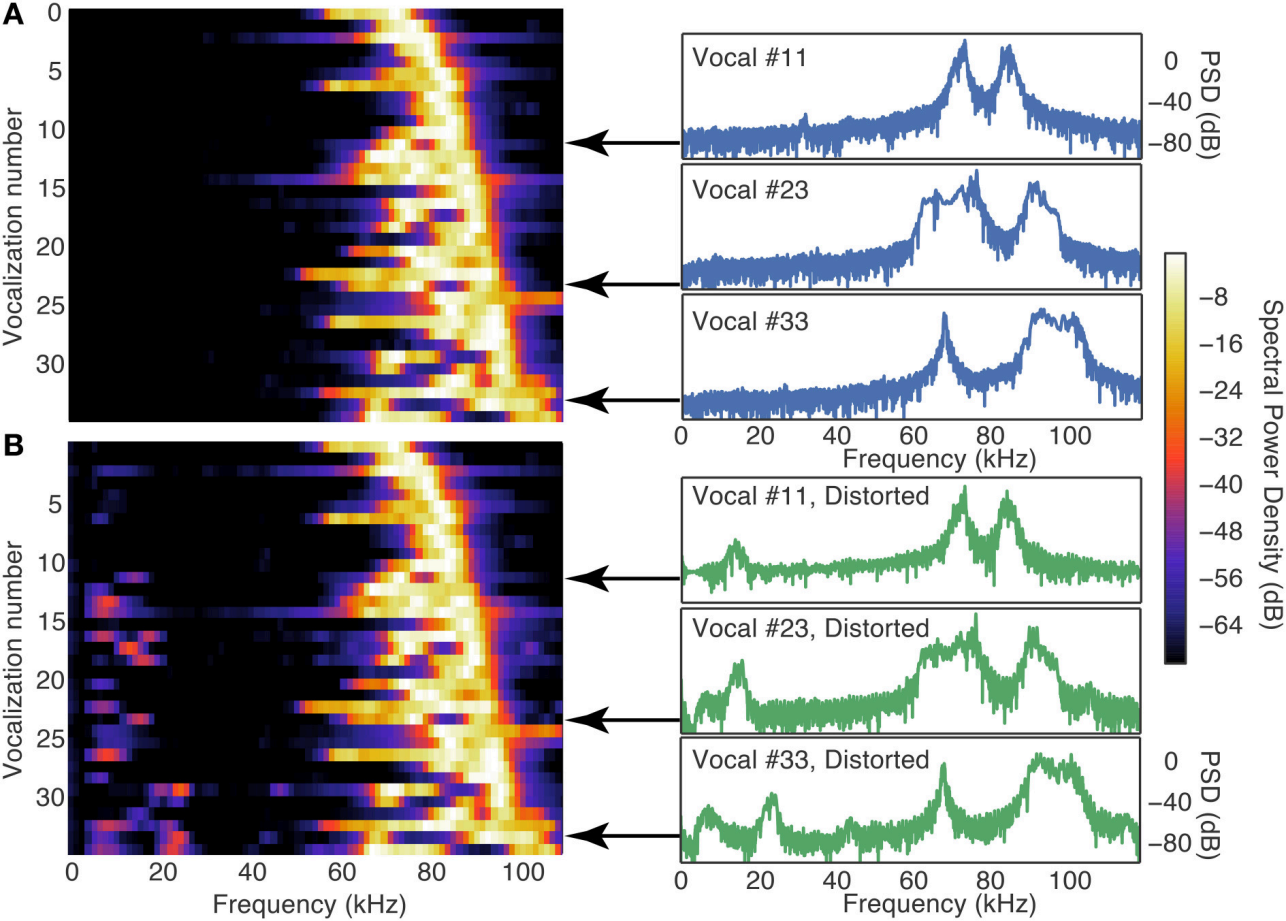
**The cochlea distortion filter introduced low frequency energy into the vocalization signals. (A)** Contour plot of the power spectral densities of all 35 vocalizations lined up in order of the frequency of their peak power (left column) and three example vocalizations with high resolution. **(B)** Same as **(A)**, but for the cochlear distortion model applied to the vocalizations. The color scale shows the spectral power relative to the maximum SPL of the stimulus (65 SPL).

**Figure 5:**
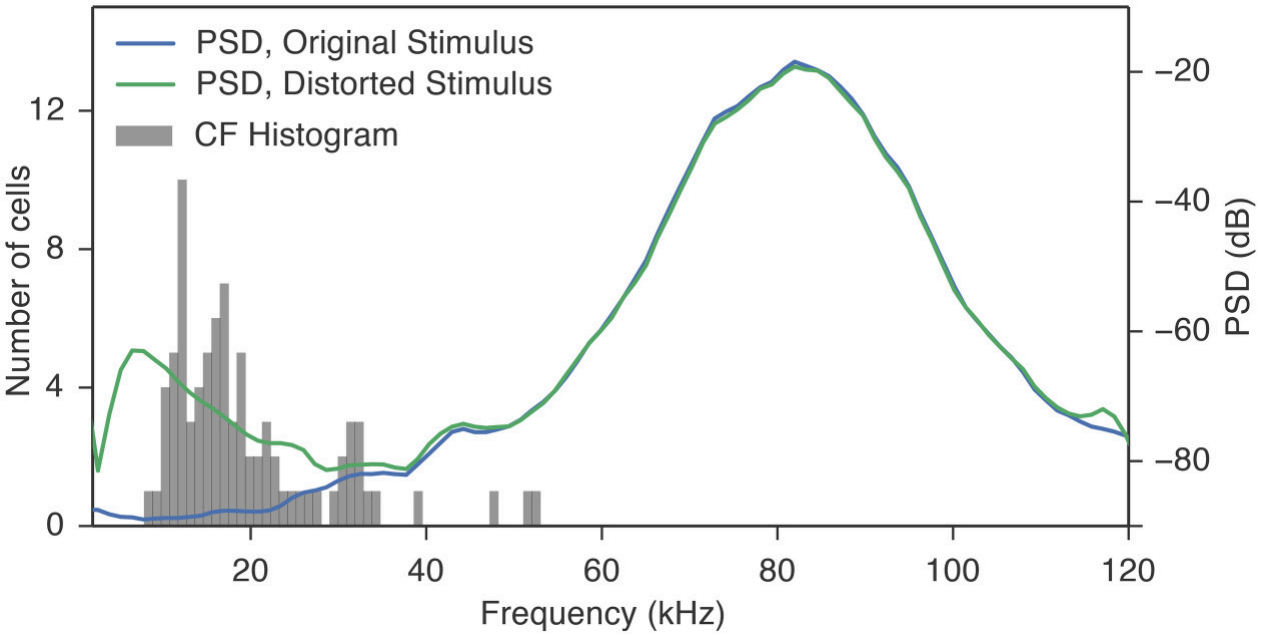
**The distortion products introduced by the cochlea distortion filter had energy (10–35 kHz) that overlapped the CFs of the fusiform cells**. The gray histogram shows the distribution of CFs of the fusiform cells. The blue trace is the average power spectral density (PSD) of all the 35 vocalization stimuli used in this study. Because the spectral power is below the response thresholds of the cells at their CF, it is unlikely that these cells were responding to the energy contained in the stimulus. The green trace is the average PSD of the same vocalization stimuli processed through the cochlea distortion filter. The majority of the CFs fall in the region where the distortion products contribute to the spectral energy.

To determine whether distortion products in the cochlea generated by the ultrasonic vocalizations contributed to the responses in the DCN, as we have shown to occur in the IC (Portfors and Roberts, 2014), we developed a phenomenological model to simulate the effective frequency spectrum of vocalizations in the presence of distortion products. We passed our vocalization stimuli through this cochlear distortion filter to create a signal that more accurately represented the signal leaving the cochlea and entering the central auditory system. The effect of the cochlea distortion filter on our suite of vocalizations is shown in Figure 4. The left panels show a heat map of the power spectral densities of all vocalizations where each horizontal strip is a different vocalization. The right panels show three examples of the power spectral densities as traces. In the natural vocalizations, there was no spectral energy below 50 kHz (Figure 4A). The distortion filter, however, introduced spectral energy below 50 kHz (Figure 4B) because of the combinations of frequencies in the ultrasonic vocalizations. The reverberations caused frequency jumps and rapid frequency sweeps in the vocalizations to overlap, and the Boltzman model generated the distortions products. This low frequency energy matched the CFs of the fusiform cells that responded to the high frequency vocalizations (Figure 5). Thus, the cochlea distortion filter provides an explaination for how fusiform cells can respond to vocalizations that have spectral energy far above the neuronal CFs.

**Figure 6:**
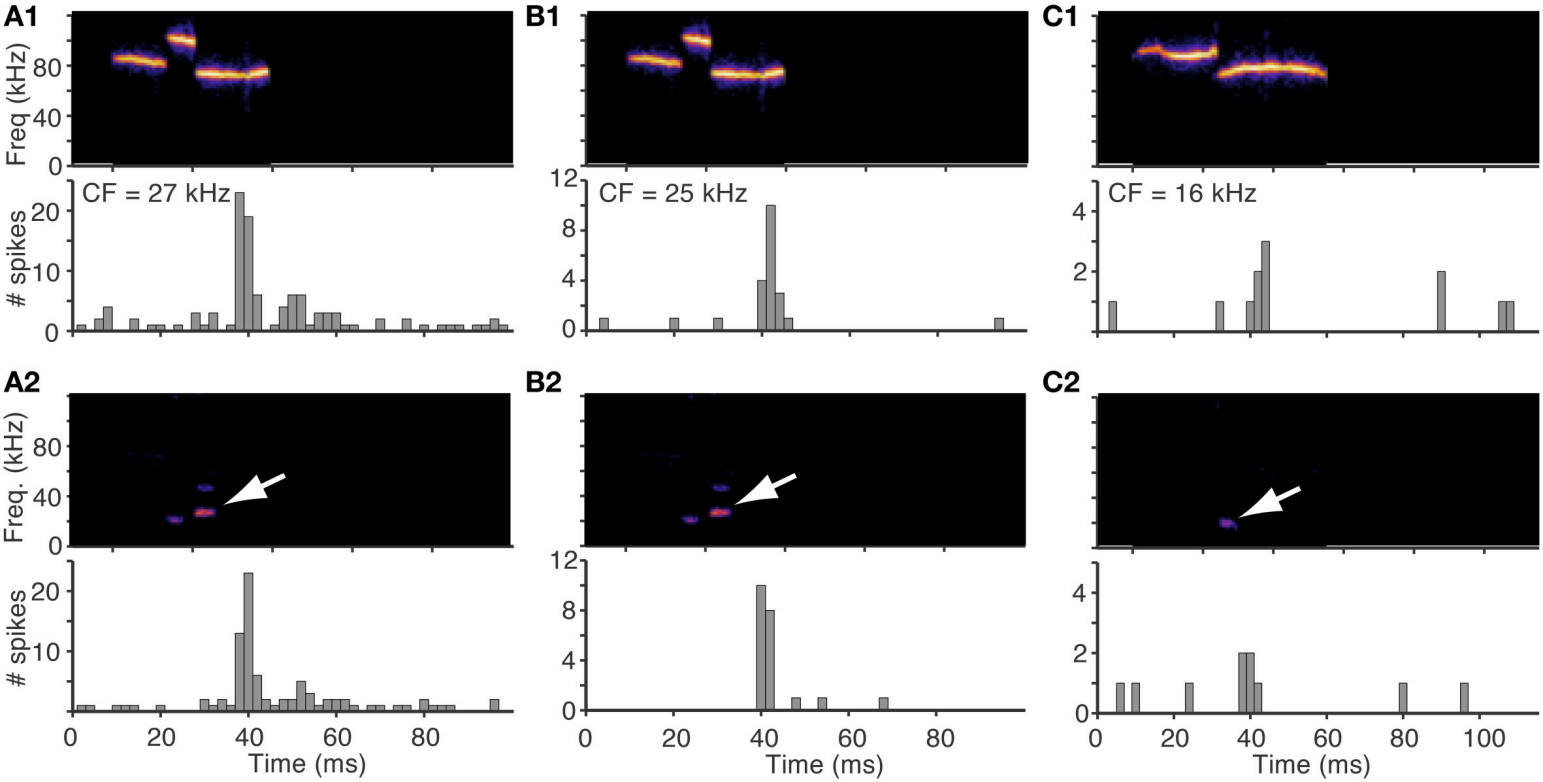
**Responses to vocalizations can be explained by responses to distortions products**. Responses of three fusiform cells to an original vocalization and to the same vocalization after being preprocessed by the cochlea distortion filter. In all cases, the histograms for the original and distorted stimuli are very similar, suggesting that the responses to the vocalizations were evoked by the low frequency distortion products. White arrows show the distortion products. The distorted signals were low pass filtered at a cutoff of 40 kHz so that only the distortion products were in the stimulus. **(A1,A2)** A fusiform cell with CF = 27 kHz **(B1,B2)** A fusiform cell with CF = 25 kHz that responded to the same vocalization as in **(A)**. **(C)** A fusiform cell with CF = 16 kHz that responded to a different vocalization.

To test this experimentally, we compared responses evoked by natural vocalizations (high pass filtered at 40 kHz to ensure no low frequency energy was present in the signal) with those evoked by the same vocalizations preprocessed through the cochlea distortion filter. The distorted signal was low pass filtered at 40 kHz so only the low frequency distortion products were presented in the signal. The similarity in both timing and intensity of neural responses evoked by these two sets of stimuli (Figure 6) confirm that the low frequency distortion products alone are driving the responses of the fusiform cells. In Figures 6A1,A2,B1,B2, the same vocalization was presented to two different cells with slightly different CFs (Figures 6A1,A2, CF = 27 kHz; Figures 6B1,B2, CF = 25 kHz). A distortion product is designated by the white arrow that is near the CF of both cells and was likely the source of the evoked responses.

The cell in Figure 6A1 responded to the natural vocalization with precisely timed spikes followed by an after-depolarization of spikes, and the response to just the low frequency distortion product showed the same spike timing pattern (Figure 6A2). The cell in Figure 6B responded with a single spike to both the natural vocalization and the distortion product with the same latencies. To quantify the similarity between the responses to the vocalizations (Figures 6A1,B1) and the responses to the distortion products (Figures 6A2,B2), we applied a similarity measure that correlates the timing of spikes to yield a rate-independent measure of temporal similarity (Holmstrom et al., 2010). The similarity between Figure 6A1 and Figure 6A2 was *S*(*A*1, *A*2) = 0.49, and the similarity between Figure 6B1 and Figure 6B2 was *S*(*B*1, *B*2) = 0.50. For comparison, the similarity measures were the same when the response of Figure 6A1 was compared to itself *S*(*A*1, *A*1) = 0.50, and lower when the responses of the two different cells were compared; *S*(*A*1, *B*1) = 0.36, and *S*(*A*2, *B*1) = 0.40. The cell in Figures 6C1,C2 had lower frequency tuning (CF = 16 kHz) and responded to a different vocalization with a lower frequency distortion product that matched the tuning of the cell. Both the natural vocalization and the distortion product evoked a burst of spikes followed by an after-hyperpolarization that inhibited spontaneous spikes for 30 ms (*S*(*C*1, *C*2) = 0.53). All of these examples clearly illustrate that the responses to the vocalizations were caused by the low frequency distortions matching the frequency tuning of the cells.

**Figure 7:**
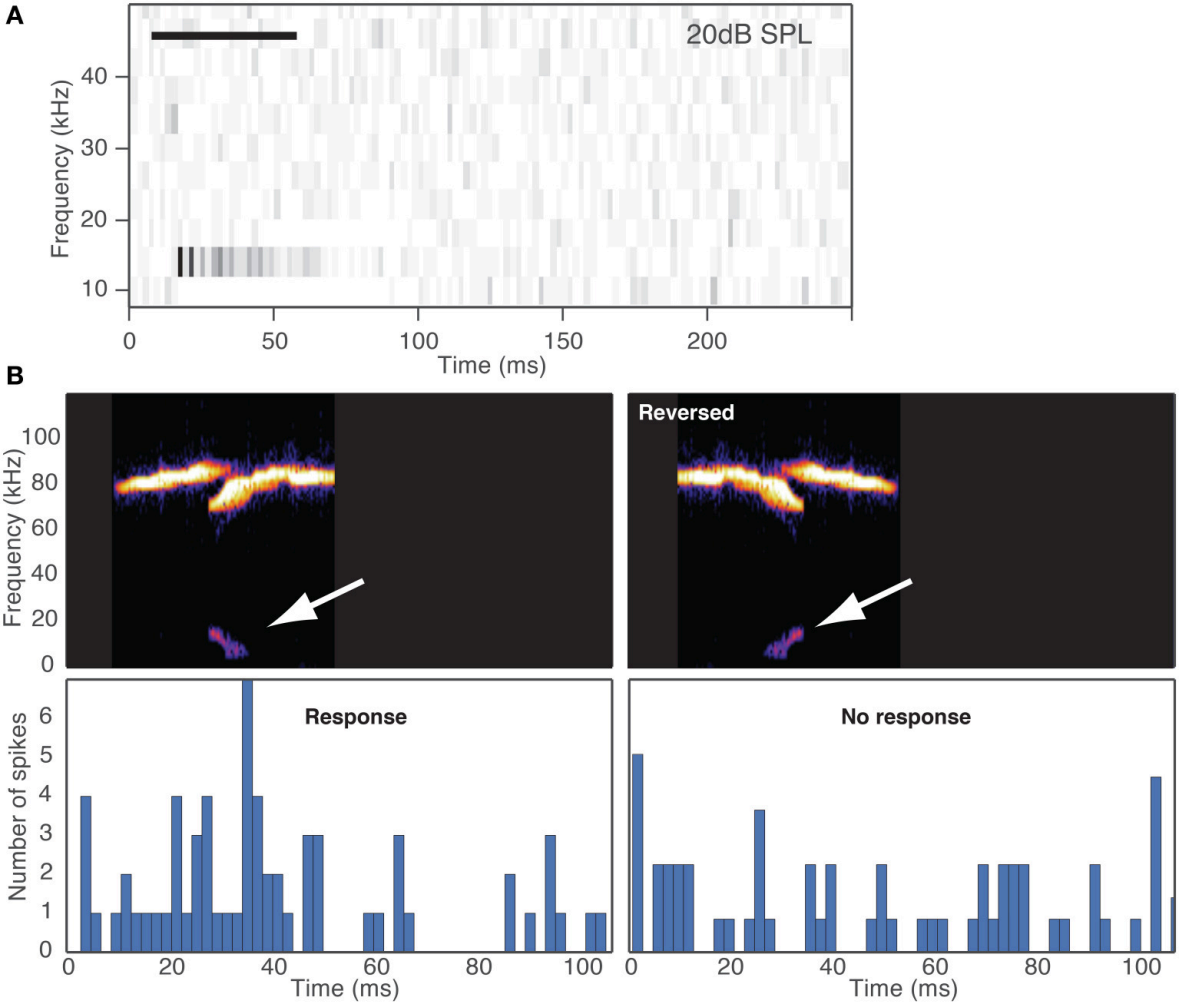
**Selectivity depends on the spectral-temporal structure of the distortion product. (A)** Spectral-temporal histogram shows responses to 11 tones (10–50 kHz) where both frequency and temporal information are displayed with time on the x-axis and tone frequency on the y-axis. Each row represents the peristimulus time histogram for the corresponding frequency. Inhibition is apparent at 10 and 18 kHz, and excitation at 14 kHz. Horizontal bar depicts the duration of the tone. **(B)** Responses of a fusiform cell to distortion products generated by the original vocalization and the same vocalization time-reversed. White arrows show the distortion products. The resulting distortions were a downsweep in the original vocalization and an upsweep in the time-reversed signal. The distortion products were presented with a 40 kHz lowpass filter. The neuron responded to the original downsweep distortion product but since the upsweep entered the inhibitory sideband first, it did not evoke a response.

Some of the cells were highly selective to some of the vocalizations in our stimulus suite while other cells responded to many of the vocalizations. One way to create selective responses is by inhibition. Inhibitory sidebands could create selectivity based on the frequency and temporal features of the distortion products. For example, if a distortion product has a temporal structure that passes through a range of frequencies, such as a down sweep, then that stimulus may evoke an inhibitory response that suppresses a potential excitatory response. The result could be that the neuron does not respond to the vocalization. Support for this hypothesis is shown by the example in Figure 7. This neuron had a CF = 12 kHz, had inhibitory sidebands (Figure 7A), and the inhibition at frequencies below the CF outlasted the duration of the stimulus. When we presented a vocalization with a down sweep distortion product with frequencies that fell within the frequency tuning curve of the neuron (Figure 7B left panel), the vocalization evoked a response. However, when we time-reverse the vocalization, the up sweeping distortion product began within the low frequency inhibitory sideband so that a response was not evoked (Figure 7B right panel). This result suggests that selectivity of fusiform cells in the DCN could depend on the full spectral-temporal structure of the distortion products. Future experiments will be important to fully explain the mechanisms underlying selectivity to vocalizations in the DCN.

## 4. Discussion

The present results show that fusiform cells in the mouse DCN respond to vocalizations in a heterogeneous manner and in a manner that is not explained by the neuronal frequency tuning properties. This provides evidence that there is some level of selectivity to vocalizations in the DCN in that neurons with similar tuning properties respond to the same vocalizations in different ways. The importance of this result is that different vocalizations can be represented by a different populations of neurons (Klug et al., 2002). Finding heterogeneous and selective responses to vocalizations in the DCN is significant because selectivity to vocalizations has historically been thought to be a property of primary auditory cortex and higher centers (Wollberg and Newman, 1972; Glass and Wollberg, 1983; Wang et al., 1995; Wang and Kadia, 2001). Several studies however, have suggested that selectivity to vocalizations emerges in the auditory midbrain (Klug et al., 2002; Portfors, 2004; Andoni et al., 2007; Portfors et al., 2009; Holmstrom et al., 2010; Portfors and Roberts, 2014), and our results now suggest that selectivity to vocalizations may actually emerge in the cochlear nucleus. Future experiments will be necessary to understand how the selectivity to vocalizations observed in the DCN and higher structures is used behaviorally for discriminating sounds.

Our findings suggest that neural circuitry in the DCN enables the beginning of selectivity to complex sounds at one of the earliest stages of processing in the central auditory pathway. In this study we focused on fusiform cells because these neurons receive inputs from multiple sources such that their output does not reflect auditory nerve input, they (along with giant cells) are the output neurons of the DCN, and they provide substantial direct input to the IC (Ryugo et al., 1981; Ryugo and Willard, 1985). Fusiform cells receive direct input from auditory nerve afferents and inputs from parallel fibers. Parallel fibers originate in the granule cell domain and synapse onto fusiform, cartwheel and giant cells in the superficial layer of DCN. The granule cells and their parallel fiber axons convey information from a wide range of auditory and non-auditory sources (Brown et al., 1988; Caicedo and Herbert, 1993; Golding et al., 1995; Weedman and Ryugo, 1996; Li and Mizuno, 1997; Schofield and Cant, 1999; Shore et al., 2000; Ohlrogge et al., 2001; Haenggeli et al., 2005). In addition, fusiform cells receive inhibitory input from cartwheel cells, whose cell bodies reside in the molecular layer (Berrebi and Mugnaini, 1991). Cartwheel cells have complex frequency tuning (Roberts and Portfors, 2008) that may arise from parallel fiber inputs, stellate cells in the molecular layer that receive electrical contact from fusiform cells or possibly auditory nerve input. Considering this complex frequency tuning and that some cartwheel cells respond to vocalizations, (Roberts and Portfors, 2008) the inhibitory input of cartwheel cells onto fusiform cells could shape how fusiform cells respond to different vocalizations. The caveat to this however is that often the response latencies to the vocalizations that we observed are too short to be driven by cartwheel cell input. The role cartwheel cells play in shaping response properties of fusiform cells requires further study with stimuli of longer duration.

The selective and heterogeneous responses to vocalizations that we observed in some fusiform cells could be explained by the known strong inhibitory sidebands that sharpen the frequency tuning curves of some fusiform cells (Young and Davis, 2002). Such frequency tuning curves can increase selectivity to vocalizations by only allowing a neuron to respond to stimuli with spectral content within the narrow excitatory frequency range or by inhibiting responses to vocalizations that contain spectral energy within the inhibitory frequency tuning curves (Portfors, 2004; Andoni et al., 2007). Pharmacological experiments in the IC that blocked glycinergic and GABAergic receptors clearly showed that selectivity decreases when inhibition is blocked (Klug et al., 2002; Mayko et al., 2012). In addition, asymmetrical inhibitory tuning around the excitatory tuning region can create selective responses to the direction of a FM sweep within a vocalization (Andoni et al., 2007). This has been well charactertized in the IC (Fuzessery et al., 2006), auditory cortex (Razak and Fuzessery, 2009), and DCN (Smith and Rhode, 1985) and we show the same effect here in the DCN in providing selectivity to distortion products generated by ultrasonic social vocalizations. For example, neurons with a low frequency inhibitory region would selectively respond to distortion products that have a FM downsweep because that sweep will pass through the excitatory high frequency region before hitting the low frequency inhibitory region. In contrast, those same neurons would not respond to a distortion product that had a FM upsweep with the same frequency bandwidth because the sweep would enter the inhibitory low frequency region of the tuning curve first and suppress any later excitatory response. In addition, inhibitory sidebands can shape selectivity to vocalizations based on the temporal pattern of the frequencies contained in the sound because sideband inhibition can continue to inhibit the cell after the stimulation has terminated (Rhode and Greenberg, 1994; Zhou et al., 2014). The complex interactions of the variety of inhibitory and excitatory inputs onto fusiform cells could create the heterogeneous responses we observed in the DCN.

As also occurs in the IC (Portfors et al., 2009; Holmstrom et al., 2010; Portfors and Roberts, 2014), neurons in the DCN often responded to ultrasonic vocalizations that had spectral energy much higher than their frequency tuning responses. We have previously suggested that these low tuned neurons in the IC respond to the cochlear distortions on the basilar membrane created by combinations of ultrasonic tone frequencies (Portfors et al., 2009; Portfors and Roberts, 2014). Responses of low frequency neurons in the IC to high frequency vocalizations are better predicted when the stimuli are processed with a nonlinear transduction model of the cochlea that generates distortion products (Lukashkin and Russell, 1998, 1999). In the current study, by processing the vocalization stimuli through a dynamic version of the nonlinear transduction model of the cochlea and then low pass filtering the stimuli, we were able to show that neuronal responses to just the distortion products were similar to the same neuron's responses to the natural vocalizations. Thus, fusiform cells are likely responding to cochlear distortion products and not the actual high frequencies in the vocalizations. Because the distortions are generated in the cochlea, our results here do not suggest that responses in IC to vocalizations are generated in DCN, but that cochlear distortions are likely the source of these responses in both nuclei. The evidence that neurons in both the DCN and IC of rodents utilize distortion products (Portfors et al., 2009; Portfors and Roberts, 2014; Gao et al., 2015) created by complex frequency interactions in vocalizations significantly alters the way we view mechanisms of auditory processing. Interestingly, distortion products have also been implicated in pitch perception of spectrally complex sounds (McAlpine, 2004).

The finding of selectivity to vocalizations in the DCN extends previous understanding of the functional role of the DCN. Research in the cat has pointed to the DCN functioning as a notch detector for sound localization tasks (Young et al., 1995; Davis et al., 1996b; Ding et al., 1999; May, 2000; Young and Davis, 2002; Oertel and Young, 2004; Reiss and Young, 2005; Zheng and Voigt, 2006). There is also evidence from lesion studies that localization in the vertical plane requires the DCN (May, 2000). However, sound localization may not be a general function of the DCN in all mammals and it may not be the major function. For example, mice do not seem to use vertical notch cues for sound localization (Lauer et al., 2011). Because of its adaptive properties (Roberts and Portfors, 2008), the DCN is likely a general adaptive filter that functions to amplify behaviorally relevant features in a complex auditory environment. Adaptive filtering of sound is important for distinguishing unexpected from expected sounds including self-generated sounds (Shore, 2005), for echo suppression (Wickesberg and Oertel, 1990; Kaltenbach et al., 1993), and/or for filtering background noise. Our data suggest that the DCN may also participate in low level discrimination of complex sounds by having fusiform cells that can distinguish between behaviorally relevant vocalizations. The mechanisms for selectivity that we have identified would not cause fusiform cell responses to change depending on the vertical location of the sound source. This is because the frequencies of the vocalizations are higher than the frequency notches caused by the head-related transfer function (Lauer et al., 2011), and the lower frequency distortion products are effectively generated in the cochlea, *after* filtering by the head-related transfer function.

Understanding the role of the DCN in auditory processing is essential because the DCN output helps shape responses to auditory stimuli in the IC. The DCN projects directly to the IC and it has been suggested that some IC responses to simple stimuli are a result of this direct input without any further shaping by additional inputs (Ramachandran et al., 1999). The results shown here suggest that at least some of the heterogeneous and selective responses to vocalizations observed in the IC could be a result of direct input from the DCN. However, considering that some responses to vocalizations are also clearly shaped and perhaps created in the IC (Klug et al., 2002; Xie et al., 2005; Mayko et al., 2012), it is most likely that selectivity to vocalizations can emerge through multiple processing stages from the brainstem to midbrain to cortex. Future studies directly comparing responses to vocalizations in multiple structures will be key to fully understanding how vocalizations are encoded in the central auditory system.

## Funding

This work was supported in part by the National Institutes of Health through National Institute of Deafness and Communication Disorders under Grant No. DC13414 to CVP.

### Conflict of interest statement

The authors declare that the research was conducted in the absence of any commercial or financial relationships that could be construed as a potential conflict of interest.
